# Spin waves across three-dimensional, close-packed nanoparticles

**DOI:** 10.1088/1367-2630/aaef17

**Published:** 2018

**Authors:** Kathryn L Krycka, James J Rhyne, Samuel D Oberdick, Ahmed M Abdelgawad, Julie A Borchers, Yumi Ijiri, Sara A Majetich, Jeffrey W Lynn

**Affiliations:** 1 NIST Center for Neutron Research, National Institute of Standards and Technology, Gaithersburg, MD 20899, United States of America; 2 Applied Physics, National Institute of Standards and Technology, Boulder, CO 80305, United States of America; 3 Department of Physics, Carnegie Mellon University, Pittsburgh, PA 15213, United States of America; 4 Department of Physics and Astronomy, Oberlin College, Oberlin, OH 44074, United States of America

**Keywords:** magnons, nanoparticles, neutron scattering

## Abstract

Inelastic neutron scattering is utilized to directly measure inter-nanoparticle spin waves, or magnons, which arise from the magnetic coupling between 8.4 nm ferrite nanoparticles that are self-assembled into a close-packed lattice, yet are physically separated by oleic acid surfactant. The resulting dispersion curve yields a physically-reasonable, non-negative energy gap only when the effective Q is reduced by the inter-particle spacing. This Q renormalization strongly indicates that the dispersion is a collective excitation between the nanoparticles, rather than originating from within individual nanoparticles. Additionally, the observed magnons are dispersive, respond to an applied magnetic field, and display the expected temperature-dependent Bose population factor. The experimental results are well explained by a limited parameter model which treats the three-dimensional ordered, magnetic nanoparticles as dipolar-coupled superspins.

## 1. Introduction

Magnetic nanoparticles are promising for the next generation of *in vivo* biomedical applications and high-density spintronic memories. For the former, the multimodal functionality [1–3] of fluorescence detection [4, 5], magnetic resonance imaging, magnetic relaxometry [6], magnetically-directed transport, drug delivery, site-selective coatings, and localized hyperthermic heating can be extremely powerful. Yet, the degree and morphology of agglomeration/self-assembly that may occur deep within tissues remain difficult to quantify, which can have profound impact on both efficacy and safety. Within fully ordered, three-dimensional (3D) spintronic media, it would similarly be advantageous to measure the strength of long-range dipolar coupling between elements in order to achieve an optimal balance between miniaturization and independent switching. Evidence of collective behavior within nanoparticle superlattices has been observed as enhanced phonon interactions [7], collective vibrational modes [8], partial charge transport across quantum dots [9], and long-range dipolar coupling across magnetic nanoparticles [10–12] which can suppress the superparamagetic behavior of isolated nanoparticles [13].

While there is much practical and theoretical [14] interest in characterizing magnons in 3D nanoscale systems, few experimental techniques are appropriate. Brillouin light scattering has been used to measure magnon coupling between ferromagnetic (FM) nanowires in arrays [15–17] and polarized photoemission electron microscopy has revealed that both dipole and higher order pole interactions are present in square lattices of circular islands [18], but these studies have been limited to systems of 2D order. Inelastic magnetic neutron scattering can easily penetrate deep within materials, including 3D ordered arrays and powders, yet is an intensity-limited technique, and its usage in nanoparticle systems has been infrequent. For example, inelastic neutron scattering has been successfully applied to measure magnetocrystalline anisotropy within the nanoparticles [19, 20] and to observe collective antiferromagnetic (AFM) spin precession within individual nanoparticles [20–22]. Here we use inelastic neutron scattering to demonstrate that magnetic spin waves form across a collection of 3D close-packed, ferrite nanoparticles, which are physically separated from one another by oleic acid coatings. Formation of collective spin waves arising from the nanoscale macrospins has been theoretically predicted [14], but to our knowledge magnons arising from 3D-ordered nanostructures have not been previously measured.

## 2. Results

### 2.1. Sample characterization

**Figure 1.**
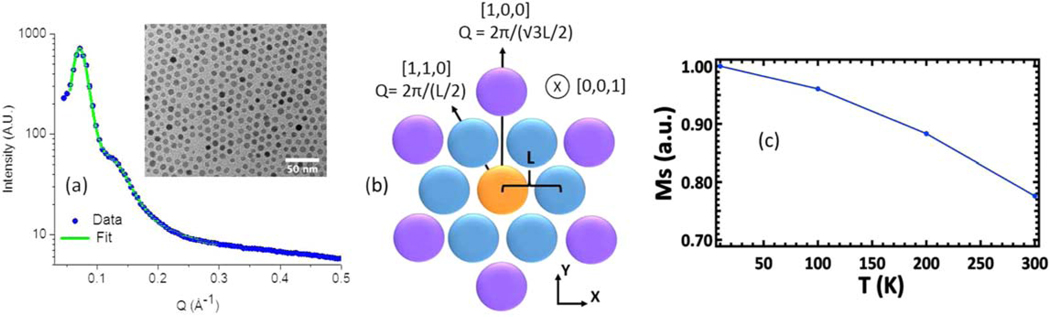
(a) SANS reveals peaks at 0.0714 and 0.129 Å^−1^ (green line fit), where error bars here and elsewhere represent one standard deviation. The inset shows a TEM image of single layer nanoparticles. (b) A close-packed plane of nanoparticles is shown. The (100) and (110) reflections in Q-space can be detected by a radial scan along the [1,0,0] and [1,1,0] directions specified by hexagonal Miller indices, respectively. Slightly offset planes are stacked along the [0,0,1] direction. (c) Magnetic saturation of a similar sample [26] as a function of temperature.

Magnetite core|manganese ferrite shell nanoparticles were prepared using the Sun method [23]. Magnetite (Fe_3_O_4_) is a common choice for biomedical applications due to its bio-compatibility; the inclusion of Mn further decreases the low magnetocrystalline anisotropy of Fe_3_O_4_ [24, 25]. Such magnetic softening could in principle promote longer ranged inter-particle magnetic coupling. However, due to differences in precursor decomposition temperatures, the particles were found to be comprised of magnetite for the innermost 6.4 nm diameter and manganese ferrite out to their surfaces [26]. The Verwey transision was not observed for the nanoparticle system based on field-cooled and zero-field cooled hysteresis measurements [26], consistent with a general trend for Verwey suppression as a function of decreasing magnetite nanoparticle size [27]. Alternating toluene and ethanol rinses were used to remove excess oleic acid and reduce incoherent hydrogen scattering, leaving a thin, protective oleic acid coat of 0.3 nm or less in thickness surrounding each nanoparticle [28]. Transmission electron microscopy images taken on thousands of nanoparticles indicates that the nanoparticle diameters are 8.4 nm ± 1.6 nm (one standard deviation), see inset of figure 1, such that the manganese ferrite shells are about 1.0 nm thick. Slow precipitation from immiscible alcohols produces face-centered cubic (fcc) supercrystallites [29], while a faster precipitation process used for this sample introduces stacking faults. The crystallites of all orientations were subsequently collected to form a powder-like sample. A close-packing arrangement was confirmed using small-angle neutron scattering (SANS) measurements performed at the NIST beamline NG7 using neutrons 6 Å in wavelength with a 11.5% wavelength spread. Taking into account the instrumental smearing [30, 31], the SANS data were fit using SasView [32]^5^ by two Lorentzians at 0.0714 and 0.129 Å^−1^, figure 1(a). These peaks most closely correspond to (100) and (110) reflections of a hexagonal close-packed (HCP) structure, figure 1(b). The (002) and (110) HCP reflections are equivalent to the (111) and (220) fcc reflections, respectively, where the (100) HCP reflection is absent in perfectly ordered fcc crystals. Thus, this sample packing can be regarded as fcc-like with truncation or stacking faults that allow the (100) HCP reflection to persist, as depicted in figure 1(b). The (100) reflection at 0.0714 Å^−1^ corresponds to the nearest-neighbor length, L, of 10.16 nm.

A similar sample of magnetite core|manganese ferrite shell nanoparticles with better inter-particle ordering, but smaller sample volume and a slightly thinner manganese ferrite shell of 0.5 nm [26], was used to obtain the temperature dependence of the magnetization (M) and to estimate the inter-particle magnetic correlation length in remanence. For this sample, SANS polarization analysis of the neutron spin before and after scattering from the sample allows the structural and magnetic scattering to be unambiguously separated [28, 33–35]. The lowest-Q, structural SANS peak at 0.0842 Å^−1^ corresponds to the typical (111) inter-particle fcc reflection, and it is well modeled as an infinite fcc paracrystal with a distortion factor of 0.12^6^ [36, 37]. These dislocations in turn broaden the (111) peak such that after instrumental resolution has been accounted Debeye–Scherrer broadening would indicate a structural coherence length of 35.5 nm ± 0.85 nm (see footnote 6) [38]. Comparison of the structural-magnetic (111) peak intensities at 1.5 T yields $\frac{M_{190\text{K}}}{M_{300\text{K}}}=1.17$ (see footnote 6), which is in close agreement to the magnetic saturation versus temperature curve of figure 1(c) produced from SQUID magnetometry measurements (see footnote 6) with a blocking temperature is about 30 K [26]. The specific temperatures of 190 and 300 K are chosen in order to normalize inelastic magnetic data acquired at these temperatures (discussed later). Finally, neutron spin-flip scattering measured at 0.0005 T and 200 K probes the near remanence, magnetic-only scattering of the nanoparticle assembly (see footnote 6). This magnetic scattering lacks the inter-particle (111) Bragg peak observed for the structural scattering and is instead well described by a fractal model of spherical nanoparticles [39, 40] whose magnetic coherence length is 19.1 nm ± 0.3 nm (see footnote 6), or roughly half that of the structural coherence. This reduction in inter-particle, FM domain size at remanence compared to inter-particle structural coherence could be due to the fact that (i) the magnetocrystalline easy-axes of the nanoparticles are randomly oriented in space and/or (ii) both FM and AFM inter-particle interactions are expected for close-packed nanoparticle arrangements [41].

### 2.2. Inelastic scattering

**Figure 2.**
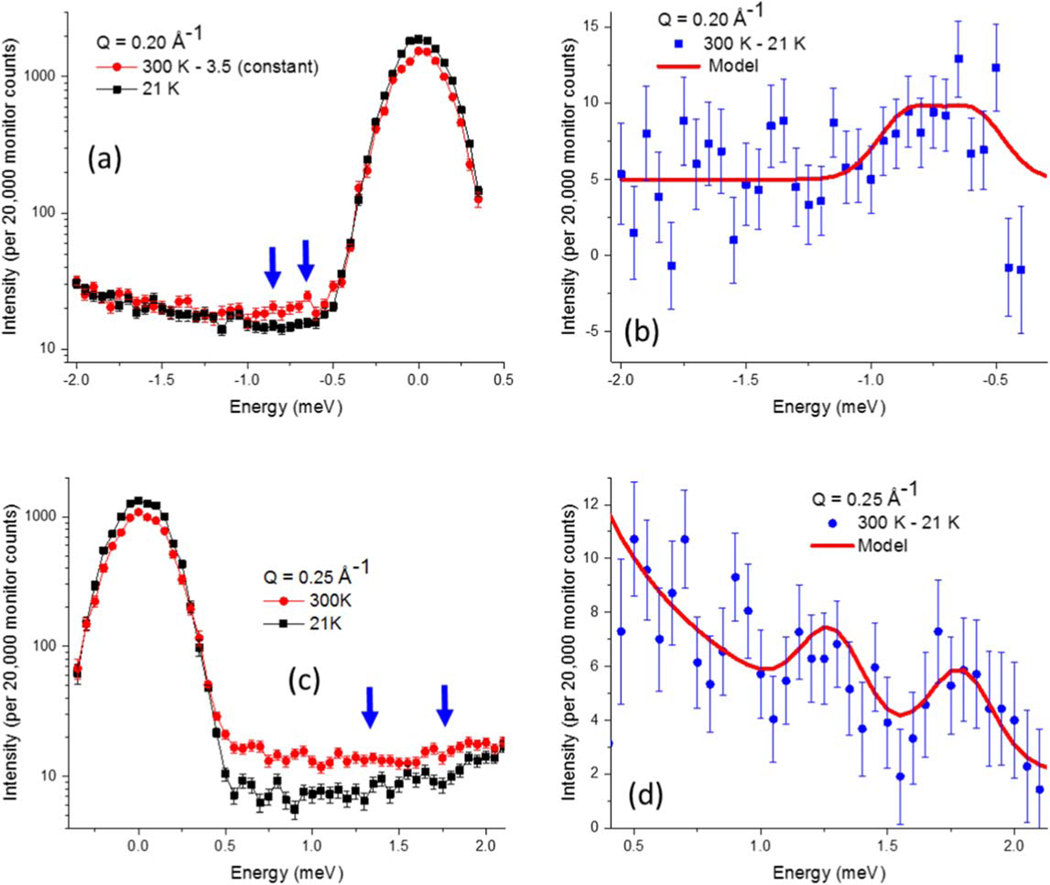
Raw data at 21 and 300 K are shown for (a) $Q=0.20\;{\text{Å}}^{-1}$ and (c) 0.25 Å^−1^, where the blue arrows highlight the local differences. The corresponding differences are shown for (b) $Q=0.20\;{\text{Å}}^{-1}$ and (d) 0.25 Å^−1^. The parameters used for the solid red curve models are listed in table 3.

**Figure 3.**
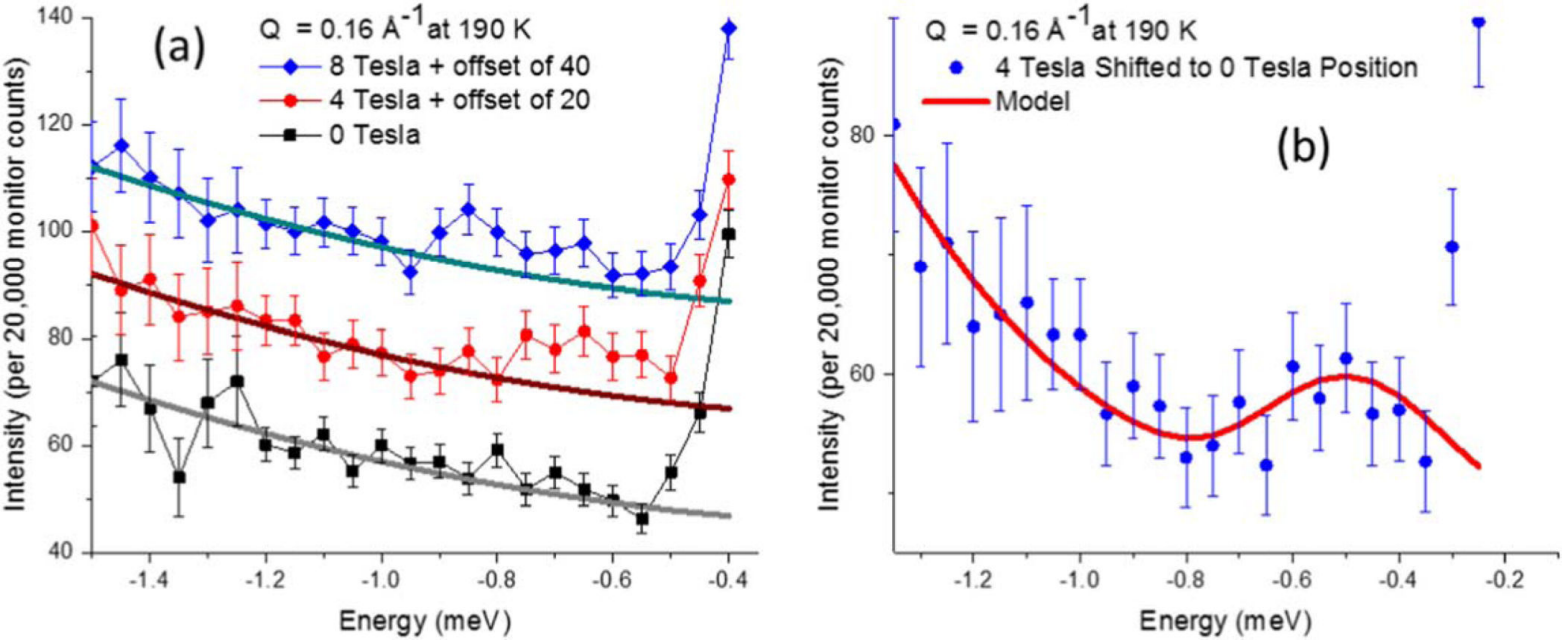
(a) Inelastic scattering at $Q=0.16\;{\text{Å}}^{-1}$ was measured at magnetic fields of 0, 4, and 8 T at 190 K with similar background slopes. The experimental 0 T excitation is not directly accessible since the inelastic peak is buried in the quasi-elastic scattering around Q=0. However, since the Zeeman shift in energy is linear with applied field, the 0 T equivalent data (b) is determined by shifting the 4 T data down in energy magnitude by the 0.14 meV difference between the 8 and 4 T peaks to 0.50 meV. The parameters used for the red line model are listed in table 3.

Inelastic neutron scattering was performed on the triple-axis instrument, BT7 [42], at the NIST Center for Neutron Research on 0.87 g of magnetite|manganese ferrite nanoparticles, characterized in figure 1(a). A vertical focusing monochromator with 25–10-10–25 min FWHM collimation was employed at an incident energy of 13.7 meV, producing an elastic energy resolution of 0.2 meV FWHM. The weak magnon peaks are most reliably extracted as a difference in temperature or applied magnetic field. Since the magnon intensity diminishes with decreasing temperature due to the Bose population factor [43], 21 K data were used to remove incoherent background scattering from 300 K data at momentum transfers of 0.20 and 0.25 Å^−1^, figure 2. Within the broad quasi-elastic peak centered around zero energy transfer, the 21 K scattering dominates the 300 K scattering, such that only peaks outside the elastic tail region are accessible. Thus, for lower energy transfers that fall within this quasi-elastic region, such as at 0.16 Å^−1^ in figure 3, an applied magnetic field of up to 8 T was used to shift the spin wave energies by the Zeeman energy.

Neutron energy gain and loss transfers probe the same excitations. The energy loss mode produces lower background scattering and was used to measure the 0.16 and 0.20 Å^−1^ data. The energy gain mode allows a slightly larger energy range to be sampled within the kinematic energy–momentum limits [44] and was used to measure the 0.25 Å^−1^ data, but it suffers from lower intensity relative to background due to the Bose population factor. The degree to which the extracted peaks are statistically significant are evaluated with both Akaike Information Criteria [45] and Bayesian Information Criteria [46] tests (see footnote 6). Although individual magnon peaks suffer from significant statistical noise, in aggregate they are statistically relevant and they can be described by an inter-particle dipole–dipole model (red curves of figures 2 and 3) that will be discussed subsequently.

**Figure 4.**
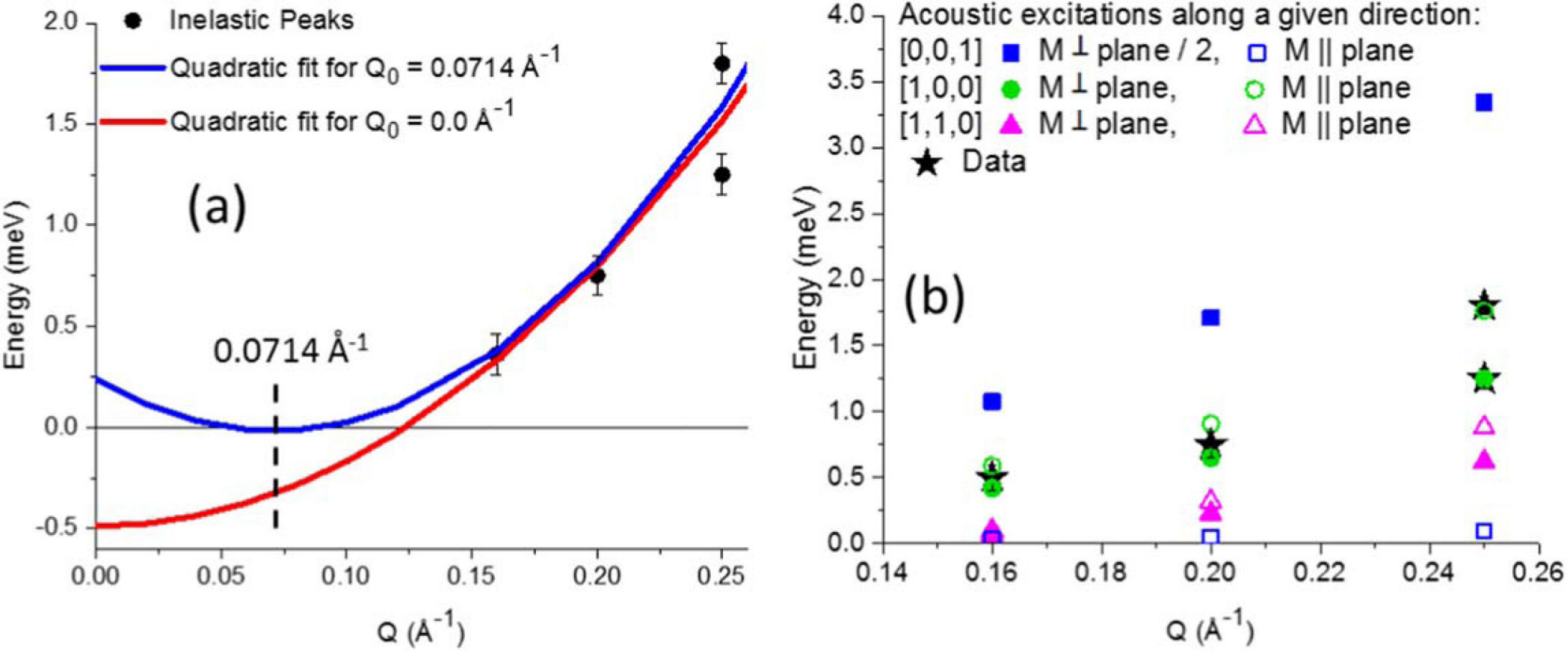
(a) Dispersion curves are obtained from the inelastic peaks, black circles taken from figures 2 and 3, using $Q_{0}=0$ (red curve) and $Q_{0}=0.0714\;{\text{Å}}^{-1}$ (blue curve). (b) Experimentally observed excitations are shown as black stars. Otherwise, the solid and open symbols correspond to theoretical acoustic FM excitations with M⊥ plane and M∥ plane, respectively, where coupling between the central nanoparticle and all n.n., all n.n.n., and 3 of the n.n.n.n. is assumed. Excitations with $\hat{Q}$ along [1,1,0] (triangles) and [0,0,1] (squares) do not fit the data, while excitations with $\hat{Q}$ along [1,0,0] (circles) describe the data well, including a splitting between M⊥ plane and M∥ plane energies with increasing Q.

Specifically, the 300 K minus 21 K inelastic scattering at $Q=0.20\;{\text{Å}}^{-1}$ yielded a weak peak at ≈0.75 meV, figures 2(a), (b). At $Q=0.25\;{\text{Å}}^{-1}$, temperature-dependent inelastic peaks were observed at 1.80 meV and 1.25 meV, figures 2(c), (d). Note that the highest-energy peak of 1.8 meV at 0.25 Å^−1^ was just within the upper bound of the observable energy range for the energy–momentum limits of this Q [44], which is why neutron energy gain mode was utilized. Conversely, for magnons of the lowest accessible energies, a horizontal-field superconducting magnet was used to shift the spin wave energies by the Zeeman energy, enabling a measurement at 0.16 Å^−1^. The observed energy shift of the inelastic excitation with an applied field additionally confirms that the observed feature is a magnon. Fields of 8, 4, and 0 T all have the same inelastic background, as shown in figure 3(a). The 8 and 4 T data show a peak shift of (0.14 ± 0.02) meV (see footnote 6). Given that the Zeeman shift is linear with applied field, the effective 0 T data shown in figure 3(b) were obtained by shifting the 4 T data down in energy by 0.14 meV. The data in a magnetic field were collected at 190 K, rather than 300 K, due to constraints on helium consumption. Dipole-based excitation energies in mean-field theory are $\propto M^{2}$, thus, the excitation energy should decrease by a factor of ${\left(\frac{M_{300\text{K}}}{M_{190\text{K}}}\right)}^{2}\approx 0.73$, discussed above, such that the observed 0.50 meV peak at 190 K would be ≈0.36 meV at 300 K. This temperature correction allows all the data to be plotted on the same dispersion curve, figure 4(a).

The spin stiffness, D, of a centrosymmetric, FM magnon can be described by the dispersion relationship $\text{Δ}E=E_{0}+Dq^{2}$. $E_{0}$ is the non-negative energy gap associated with magnetocrystalline anisotropy of the material. For this system, $E_{0}$ is negligible as evidenced by the dispersion curves of bulk Fe_3_O_4_ [47] and bulk MnFe_2_O_4_ [48], which means that the magnetocrystalline anisotropy is small. Plotting the primary inelastic peaks at 300 K of (0.36, 0.75, 1.25, 1.8) meV located at Q values of (0.16, 0.20, 0.25, 0.25) Å^−1^ yields a non-physical negative energy gap of −0.51 ± 0.41 meV, red curve figure 4(a). However, if the instrumental Q is shifted by an amount $Q_{0}$,

(1) $$q=Q-Q_{0},$$

this unphysical result is removed. Specifically, setting $Q_{0}$ equal to the primary reflection at $Q=0.0714\;{\text{Å}}^{-1}$ (figure 1(a)) produces the expected, near-gapless dispersion with an effective spin stiffness D of 51 meV Å^2^ ± 12 meV Å^2^ (one standard deviation), blue curve figure 4(a).

The experimentally observed $Q_{0}$ shift of 0.0714 Å^−1^ is most readily explained as renormalization of q by nearest reciprocal-lattice vector [49, 50] arising from the well-defined nanoparticle-to-nanoparticle spacing along $\hat{Q}=[1,0,0]$ and equivalent directions, where the dominant interaction between nanoparticles would be dipolar coupling. If this measured dispersion were based on atomic exchange instead, the measured D would be about 17% that the spin stiffness of bulk Fe_3_O_4_ [47] and 29% the spin exchange stiffness of bulk MnFe_2_O_4_ [48]. If the exchange stiffness of bulk MnFe_2_O_4_ was assumed, the minimum Q that could be observed from an intra-particle excitation within an 8.4 nm diameter nanoparticle would be $1.42\times 2\pi /84\;\text{Å}=0.106\;{\text{Å}}^{-1}$ [20] with an energy of 1.95 meV, which is outside the experimentally accessible limits [44] and explains why we do not observe the dispersion from spin exchange stiffness. Moreover, the large required Q-shift by itself precludes the excitation from being associated with typical intra-particle exchange. In addition, our 8.4 nm nanoparticles reside in a truncated fcc-like arrangement with nanoparticle-to-nanoparticle distance of L=10.16nm, table 1, resulting in an average nanoparticle-to-nanoparticle gap of 1.76 nm filled by oleic acid and air. Such physical separation is unlikely to allow any appreciable inter-particle atomic exchange coupling which, using Fe_3_O_4_ as example, should be highly localized and based primarily on the existence of nearest and next-nearest neighbors within 0.9 times the unit cell of 0.84 nm = 0.76 nm [51]. Finally, while substantial Q-shifts can occur from Dzyaloshinskii–Moriya intra-particle interactions [52], the associated Q-shift [53] of 0.21 Å^−1^ (see footnote 6) determined from the ratio of exchange stiffness to Dzyaloshinskii–Moriya interaction strength [26, 54] does not fit the data.

### 2.3. Inter-particle magnon model

Generally, both atomic super-exchange and dipolar interactions need to be considered when evaluating inter-nanoparticle magnetic interactions [14]. However, the inter-nanoparticle coupling of this particular system should be dipolar, rather than exchange based, as discussed above. Excitations arising from a simplified approximation of dipole–dipole coupled, magnetic nanoparticles in an ordered array can be treated with the same formalism that is used for exchange-coupled atomic spins in a lattice [55], where $Q_{0}$ of equation (1) is the nearest inter-particle reciprocal-lattice vector rather than the nearest atomic reciprocal-lattice vector [49, 50]. Specifically, the resulting dispersion can be calculated using general spin wave formalism [56] where $J_{j}S$ is any energy by which a central object, i, and its neighbors j, are coupled:

(2) $$\hbar \omega A_{i}=\sum_{j}J_{j}S\left(A_{i}-A_{j}{\text{e}}^{\text{i}q\cdot \left[R_{j}-R_{i}\right]}\right).$$

R refers to the relative spatial position of objects i and j, while $A_{i}$ and $A_{j}$ are the wave function amplitudes of a phonon or magnon excitation. If $A_{i}=A_{j}$, the oscillation is an acoustic Goldstone mode with a zero energy gap at q=0; if $A_{i}=-A_{j}$ the oscillation is an optic mode with an energy gap ≫0. Note that the model assumes a quasi-infinite, HCP-like lattice of nanoparticles where at remanence the domain wall boundaries between magnetic clusters are not well-defined, but instead are gradual [57]. Such a structure should produce spin waves, in contrast with finite clusters which would produce discrete, q-broadened eigenmodes [55]. This distinction holds true even when the magnetic coupling is only considered for the nearest-neighbor nanoparticles [55]. The fact the q-width of the observed magnons (≈0.28 meV) is (a) close to the instrumental resolution of 0.20 meV and (b) similar for both low and high applied magnetic fields (where the spins are certain to have long-range coherence), strengthens this interpretation.

To calculate the dipole-based $J_{j}S$ values, the innermost 6.4 nm diameter is assumed to be composed of Fe_3_O_4_ and the remaining 1.0 nm of shell is assumed to be MnFe_2_O_4_ [26]. At room temperature, the magnetization, M, of bulk Fe_3_O_4_ and MnFe_2_O_4_ are 4.76 × 10^5^ A m^−1^ [58] and 4.12 × 10^5^ A m^−1^ [59], respectively. The relative nanoparticle positions are taken from table 1 where the maximum distance of $\sqrt{(}3)L$ is chosen to match the maximum magnetic coherence over which dipole–dipole coupling is calculated (see footnote 6). If the nanoparticle-to-nanoparticle spacing is ⩾ three nanoparticle diameters, the spherical nanoparticles can be treated as point dipoles. However, due to the close proximity of the nanoparticles, each nanoparticle is broken into sub-unit cubes of 4 Å per side over which the dipolar calculation is performed (using even smaller sub-units does not substantially alter the results). Note the calculated $J_{j}S$ values M∥ to any direction within the plane are nearly identical and are, thus, collectively referred to as M∥ plane (see figure 1(b)). M along the [0,0,1] direction is referred to as M⊥ plane. The results are given in table 2 where +/− signs indicate a preference for FM and AFM alignment, respectively. The tendency toward AFM coupling between alternating layers with M∥ plane has been previously observed [41].

AFM coupling is not considered further since the dispersion is not linear with q (such a fit would result in a non-physical negative gap), but rather the dispersion fits the FM $E=Dq^{2}$ form, figure 4(a). Thus, neighboring nanoparticle locations for which AFM coupling is preferred are assumed to belong to a magnetic domain uncorrelated from the domain containing the central nanoparticle, j. For M∥ plane FM coupling could exist between the central nanoparticle and 1n.n., 2n.n.n., 1n.n.n.n., and 2n.n.n.n., while for M⊥ plane FM coupling could exist between the central nanoparticle and 2n.n., 2n.n.n., and 3n.n.n.n, table 2. Yet, analysis of a similar sample of magnetite core|manganese ferrite shell nanoparticles indicates that the magnetic coherence length is roughly half that of the structural coherence length (see footnote 6) at remanence which extends out to the n.n.n.n. Thus, the average number of n.n.n.n. that are magnetically correlated with the central nanoparticle is taken as a model parameter. As a starting point, the model includes the strongest magnetic coupling of the central nanoparticle with the 3n.n.n.n., and drops the weaker magnetic coupling with the 1n.n.n.n. and 2n.n.n.n., although the full range from including none to all of the n.n.n.n is evaluated.

Combining tables 1 and 2 with equation (2) allows all dipolar-based excitation values to be calculated, as shown in table 3 for $\hat{Q}$ along the [1,0,0] direction. As figure 4(b) demonstrates, M⊥ plane and M∥ plane FM excitations from $\hat{Q}=[0,0,1]$ are too small to account for the experimental data (even if all possible n.n.n.n. are included (see footnote 6)), while M⊥ plane FM excitations from $\hat{Q}=[0,0,1]$ are too large (even if none of the possible n.n.n.n. are included (see footnote 6)) and the M∥ plane excitations from $\hat{Q}=[0,0,1]$ are negligible. Optical excitations have significant energy gaps on the order of 10ʼs to 100ʼs of meV (see footnote 6), and are thus also excluded. However, acoustic, FM excitations from $\hat{Q}=[1,0,0]$, corresponding to the most prominent reflection (figure 1(a)), fit the data well. The data indicate that two excitations are present which become more clearly resolved with increasing Q, figures 2 and 3. These correspond to the lower-energy M⊥ plane (solid green circles) and higher-energy M∥ plane (open green circles) of the $\hat{Q}=[1,0,0]$ excitations, figure 4(b).

In order to plot the modeled calculations of $\hat{Q}$ in the [1,0,0] and equivalent directions from table 3 and figure 4(b) alongside the experimental data, the excitations are each represented by Gaussian peaks with a FWHM of 0.28 meV, based on the two resolved $Q=0.25\;{\text{Å}}^{-1}$ peak widths. Since this width is larger than the elastic energy resolution of 0.20 meV (where the energy resolution should narrow further for inelastic scattering), this suggests the experimental peaks are broadened due to finite size of the nanoparticle magnetic domains. Sloped backgrounds and relative peak amplitudes (for both M⊥ plane and M∥ plane excitations) of 1.0, 0.67, and 0.46 were used for the Q values of (0.16, 0.20, and 0.25) Å^−1^. However, it should be noted that the 0.16 Å^−1^ data were collected in a magnet with thin silicon windows, while the data of the remaining two Q values were collected in an Al-walled cryostat with higher neutron absorption. Small adjustments in the calculated excitation energies of (0.0, 0.06, and 0.03) meV best describe the experimental data at Q values of (0.16, 0.20, and 0.25) Å^−1^, as shown in the last columns of table 3. These adjustments can be explained by a decrease in both the instrumental Q-resolution and energy-resolution at low Q values that can shift the data up to 10% of its median energy value. The models, based on table 3 and figure 4(b), are shown as red curves in figures 2 and 3.

## 3. Discussion

The model has two parameters, M of the material (where the excitation energy is $\propto M^{2}$) and the choice of where to truncate the magnetic coupling between the central nanoparticle and n.n.n. or n.n.n.n. given that the magnetic coherence length is likely reduced from the structural coherence of about $\sqrt{(}3)L=\text{n}.\text{n}.\text{n}.\text{n}$. (see footnote 6). We have effectively eliminated the first parameter by assuming temperature-dependent, bulk M values. As shown in figure 4(b), the best fit of the second parameter (with $\hat{Q}=\text{the}[1,0,0]$ and equivalent directions) involves all the n.n., all the n.n.n., and the 3n.n.n.n., but not the 1n.n.n.n. or 2n.n.n.n which have considerably weaker magnetic coupling than the 3n.n.n.n. with the central nanoparticle (table 2).

The M⊥ plane and M∥ plane $\hat{Q}=[1,0,0]$ acoustic magnons closely match the data (figure 4(b)), including the overlap into a broad peak at Q=0.16 and 0.20 Å^−1^ which resolves into two excitations by 0.25 Å^−1^. Conversely, the acoustic magnons associated with $\hat{Q}=[1,1,0]$ and [0,0,1] peaks do not fit the data, regardless of how many n.n.n.n. are included or excluded from the model (see footnote 6). It is worthwhile to note that if a truncated HCP-like, rather than truncated fcc-like, arrangement had been used for the calculations (table 1), the results would be very similar.

## 4. Conclusions

In summary, we have observed inter-particle magnons originating from a 3D system of close-packed magnetic nanoparticles. The measured excitations follow a $Q^{2}$ dispersion which produces a physically-reasonable, non-negative energy gap only when the $Q_{0}$ is renormalized to the inter-particle Bragg position of 0.0714 Å^−1^. Thus, the magnons originate from inter-nanoparticle interactions, and not from intra-particle excitations, even though the nanoparticles are physically separated by oleic acid coatings. Additionally, the magnons display both the Bose factor temperature dependence and shift to higher energy in response to an applied magnetic field, confirming their magnetic origin. These magnons are well described by a model which treats the nanoparticles as dipole–dipole coupled superspins in an array.

The tendency toward self-assembly into close-packed 2D and 3D structures is a fairly common feature for systems of attractive, spherical particles. In ordered systems of magnetic nanoparticles, such as the one presented here, the magnons are a measure of the $J_{N}S$ dipolar coupling strength and spatial extent between nanoparticles. The presence of magnons underscores that the influence of dipolar coupling is non-trivial and induces magnetic coherence across multiple nanoparticles. In systems of undetermined morphology, including many magnetic nanoparticle-based biological applications, the measurement of such magnons could provide insight into self-assembly and chaining properties.

## Supplementary Material

Supp1

## Figures and Tables

**Table 1.** Close-packed nanoparticle locations about a central nanoparticle at (0,0,0). In location, the numeral refers to plane stacking along the [0,0,1] direction (see figure 1(b)), while n.n., n.n.n., etc refers to nearest-neighbor and next-nearest-neighbor, etc.

Distance	Location	Number	Positions in (X,Y,Z)

L	1n.n.	6	$(\pm \text{L},0,0),\left(\pm \frac{L}{2},\pm \frac{\sqrt{3}L}{2},0\right)$
L	2n.n.	6	$\left(0,\frac{L}{\sqrt{3}},\frac{L\sqrt{2}}{\sqrt{3}}\right),\left(0,-\frac{L}{\sqrt{3}},-\frac{\sqrt{2}L}{\sqrt{3}}\right),\left(\pm \frac{L}{2},-\frac{L}{\sqrt{12}},\frac{\sqrt{2}L}{\sqrt{3}}\right),\left(\pm \frac{L}{2},\frac{L}{\sqrt{12}},-\frac{L\sqrt{2}}{\sqrt{3}}\right)$
$\sqrt{2}L$	2n.n.n.	6	$\left(0,\frac{-2L}{\sqrt{3}},\frac{\sqrt{2}L}{\sqrt{3}}\right),\left(0,\frac{2L}{\sqrt{3}},\frac{-\sqrt{2}L}{\sqrt{3}}\right),\left(\pm L,\frac{L}{\sqrt{3}},\frac{L\sqrt{2}}{\sqrt{3}}\right),\left(\pm L,\frac{-L}{\sqrt{3}},\frac{-L\sqrt{2}}{\sqrt{3}}\right)$
$\sqrt{3}L$	1n.n.n.n.	6	$(0,\pm \sqrt{3}L,0),\left(\pm \frac{3L}{2},\pm \frac{\sqrt{3}L}{2},0\right)$
$\sqrt{3}L$	2n.n.n.n.	12	$\left(\pm \frac{L}{2},\frac{\sqrt{2}5L}{\sqrt{1}2},\frac{\sqrt{2}L}{\sqrt{3}}\right),\left(\pm \frac{L}{2},\frac{-\sqrt{2}5L}{\sqrt{1}2},\frac{-\sqrt{2}L}{\sqrt{3}}\right),\left(\pm \frac{3L}{2},\frac{-L}{\sqrt{1}2},\frac{\sqrt{2}L}{\sqrt{3}}\right),$
			$\left(\pm \frac{3L}{2},\frac{L}{\sqrt{12}},\frac{-\sqrt{2}L}{\sqrt{3}}\right),\left(\pm L,\frac{-2L}{\sqrt{3}},\frac{\sqrt{2}L}{\sqrt{3}}\right),\left(\pm L,\frac{2L}{\sqrt{3}},\frac{-\sqrt{2}L}{\sqrt{3}}\right)$
$\sqrt{3}L$	3n.n.n.n.	6	$\left(0,\frac{L}{\sqrt{3}},\frac{-\sqrt{8}L}{\sqrt{3}}\right),\left(0,-\frac{L}{\sqrt{3}},\frac{\sqrt{8}L}{\sqrt{3}}\right),\left(\pm \frac{L}{2},-\frac{L}{\sqrt{12}},\frac{-\sqrt{8}L}{\sqrt{3}}\right),\left(\pm \frac{L}{2},\frac{L}{\sqrt{12}},\frac{\sqrt{8}L}{\sqrt{3}}\right)$

**Table 2.** Calculated average $J_{N}S$ per nanoparticle pair at 300 K. Values can be converted to 190 K by multiplying by a factor of 1.37, discussed above. The +,− signs indicate preferred FM, AFM alignment, respectively.

Location	$J_{N}S$ for M∥ plane	$J_{N}S$ for M⊥ plane

1n.n.	11.073 meV	−11.941 meV
2n.n.	−4.250 meV	18.839 meV
2n.n.n.	0.512 meV	0.511 meV
1n.n.n.n.	1.332 meV	−2.139 meV
2n.n.n.n.	0.547 meV	−0.546 meV
3n.n.n.n.	−1.753 meV	4.031 meV

**Table 3.** Excitations along $\hat{Q}=[1,0,0]$ are calculated by combining equation (2) and table 2 (see supplmental material (see footnote 6) for details). Both M⊥ plane and M∥ plane excitations include FM interactions between the central nanoparticle and all n.n., all n.n.n., and the 3n.n.n.n.

Q (Å^−1^)	q (Å^−1^)	Temperature (K)	Calculated FM excitations of M⊥ plane M∥ plane (meV)	Fitted FM excitations of M⊥ plane, M∥ plane (meV)

0.160	0.088	190	0.42, 0.59	0.42, 0.59
0.200	0.128	300	0.65, 0.91	0.59, 0.85
0.250	0.178	300	1.25, 1.76	1.28, 1.79

## Footnotes

5 The identification of any commercial product or trade name does not imply endorsement or recommendation by the National Institute of Standards and Technology.

6 Supplemental material is available online at stacks.iop.org/NJP/20/123020/mmedia.
