# Supplementary material for: Spin waves across three-dimensional, close-packed nanoparticles
Source: New J Phys. Author manuscript; Available in PMC 2024 Nov 19. (PMC11574860; doi:10.1088/1367-2630/aaef17)
Supplement: Supp1 [file NIHMS1548450-supplement-Supp1.pdf]

Supplement: Spin Waves Across 3-Dimensional, Close-Packed Nanoparticles

Kathryn L. Krycka

NIST Center for Neutron Research, National Institute of Standards and Technology, Gaithersburg, MD 20899

E-mail: kathryn.krycka@nist.gov

James J. Rhyne

NIST Center for Neutron Research, National Institute of Standards and Technology, Gaithersburg, MD 20899

Samuel D. Oberdick

Applied Physics, National Institute of Standards and Technology, Boulder, CO 80305

Ahmed M. Abdelgawad

Department of Physics, Carnegie Mellon University, Pittsburgh, PA 15213

Julie A. Borchers

NIST Center for Neutron Research, National Institute of Standards and Technology, Gaithersburg, MD 20899

Yumi Ijiri

Department of Physics and Astronomy, Oberlin College, Oberlin, OH 44074

Sara A. Majetich

Department of Physics, Carnegie Mellon University, Pittsburgh, PA 15213

Jeffrey W. Lynn

NIST Center for Neutron Research, National Institute of Standards and Technology, Gaithersburg, MD 20899

July 2018

Submitted to: *New J. Phys.*

1. Inter-particle Structural Correlation Length

The most prominent, structural $Q = 0.0714\ \mathring{A}^{-1}$ peak for the [1,0,0] and equivalent directions, Fig. 1a of the main text, is fit using SASView [1, 2] by a Lorentzian with a full-width half-maximum of $(0.0148 \pm 0.0004)\ \mathring{A}^{-1}$ after the experimental resolution has been properly accounted for [3]. The Scherrer formula [4],

$$D_{Struc} = 0.94\lambda/\beta cos(\theta/2), \tag{1}$$

allows for an estimate of the structural coherence length, D_{Struc}. The neutron wavelength, λ, = 6 $\mathring{A}$. β is the full-width half-maximum of the SANS scattering angle, θ in radians, where $\theta = sin^{-1}(\lambda Q/2\pi)$. Together this yields $D_{Struc} = (40.0 \pm 1.1)$ nm.

In order to place this in the context of relative distances for n.n., n.n.n., and n.n.n.n., Table I. of the main text, the maximum relevant length scale is twice the n.n., n.n.n., or n.n.n.n. distance since by symmetry each nanoparticle has neighbors at both the $\pm$ positions plus the width of the outermost nanoparticles. Thus, a measured crystalline length (40.0 $\pm$ 1.1) nm includes the n.n. at $2 \times L$ + 8.4 nm = 28.7 nm, the n.n.n. at $2 \times \sqrt{(2)}L$ + 8.4 nm = 37.1 nm, and most to all of the n.n.n.n. at $2 \times \sqrt{(3)}L$ + 8.4 nm = 43.6 nm, where the nanoparticle-to-nanoparticle distance L = 10.16 nm and the nanoparticle diameter = 8.4 nm. Note that the average crystallite length observed should be an approximate average between n.n.n. and n.n.n.n. (40.4 nm) since the peak width measured per crystallite depends on its relative orientation. At magnetic fields well below saturation, however, the magnetic domains may be smaller than the structural coherence length, so $\sqrt{(3)}L$ represents the maximum distance over which magnetic correlations among nanoparticle neighbors should be considered. As will be shown in the next section, the nanoparticles can be thought of as residing in a long-range, close-packed crystal with dislocations, rather than in finite crystallites, leading to the observed reduction in structural coherence.

2. Magnetic and Coherence Measurements on Similar Sample

Here we examine the relative structural and magnetic coherence lengths, as well as temperature-dependent magnetization, from a similar sample of magnetite core|manganese ferrite shell nanoparticles with better FCC crystalline ordering, but smaller sample volume and a slightly thinner manganese ferrite shell of 0.5 nm [5]. All small-angle neutron scattering (SANS) measurements were performed using 5.5 $\mathring{A}$ neutrons with an 11.5 % wavelength spread. Polarization analysis of the neutron spin before and after scattering from the sample allows the nuclear (structural) and magnetic scattering to be unambiguously separated [6, 7, 8]. In our notation, arrows are used to indicate the neutron spin relative to the applied field, with the first and second arrows referring to the polarization state before and after sample scattering, respectively. The polarization efficiency of all the spin optics has been measured and corrected for [9].

2.1. Structural Coherence

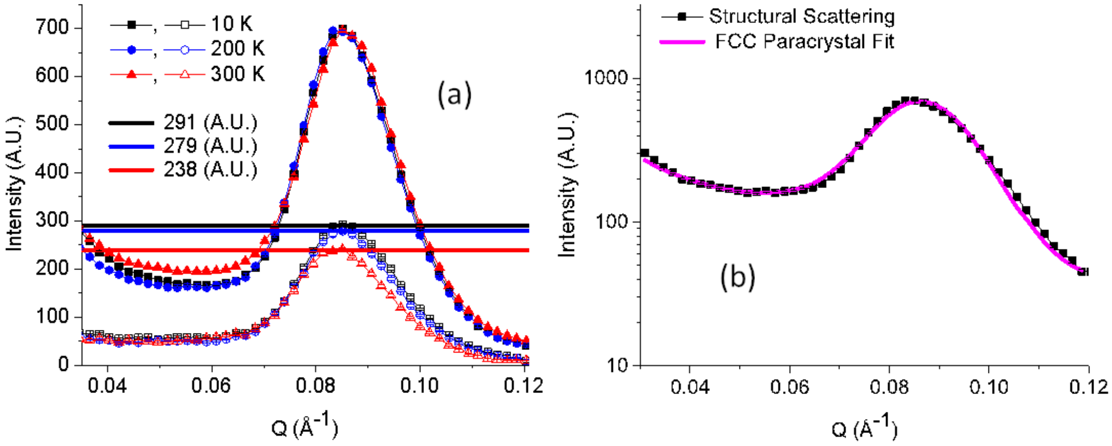

Figure 1. (a) Structural (solid points) and structural-magnetic (open points) SANS at 1.5 T are shown for a similar sample with long-range FCC order. The horizontal lines are a guide to the eye showing the maximum height of each magnetic peak. Error bars here and elsewhere represent one standard deviation. (b) Structural data (200 K data shown) can be fit by a face-centered cubic (FCC) paracrystal with a distortion factor of 0.12.

A nearly saturating, horizontal magnetic field of 1.5 T was applied to the sample such that $\downarrow\downarrow + \uparrow\uparrow$ data collected within sector cuts of $\pm$ 10° about the horizontal direction yield structural information (solid points of Fig. 1). The lowest-Q structural peak at $Q = 0.0842$ $\AA^{-1}$ corresponds to the (111) FCC reflection, Fig. 1a, and is fit using SASView [1] by a Lorentzian with a full-width half-maximum of (0.0166 ± 0.0004) $\AA^{-1}$ after the experimental resolution has been properly accounted for [3]. Following the same procedure as above [4], D_{Struc} is determined to be 35.5 nm $\pm$ 0.85 nm. Note that the ratio of D_{Struc} / nanoparticle diameter is 4.8 for both the similar and primary samples. Alternatively, the structural scattering can be well described by an quasi-infinite, face-centered cubic (FCC) paracrystal [10, 11] with a dislocation factor (*i.e.* degree of distortion of any given scattering object from its position in a perfect crystal) of 0.12, Fig. 1b.

2.2. Temperature-Dependent Magnetization

At 1.5 T, $\downarrow\downarrow - \uparrow\uparrow$ SANS data collected within sector cuts of $\pm$ 10° about the vertical direction yield twice the structural-magnetic scattering $\propto$ the net sample magnetization, M, parallel to the applied field [7] (open points of Fig. 1). The relative nuclear-magnetic peak intensity heights of 291 at 10 K, 279 at 200 K, and 238 at 300 K yield $\frac{M_{200\ K}}{M_{300\ K}} =$ 1.17, which is a good approximation for magnetic scaling between the 190 K and 300 K data.

SQUID magnetetomotry, Fig. 2, was used to generate the magnetic saturation vs. temperature curve in Fig. 1c of the main text. The increase of saturated magnetization (at 5 T) between 300 K and 190 K, based on SQUID measurements, is also about 0.90/0.77 = 1.17.

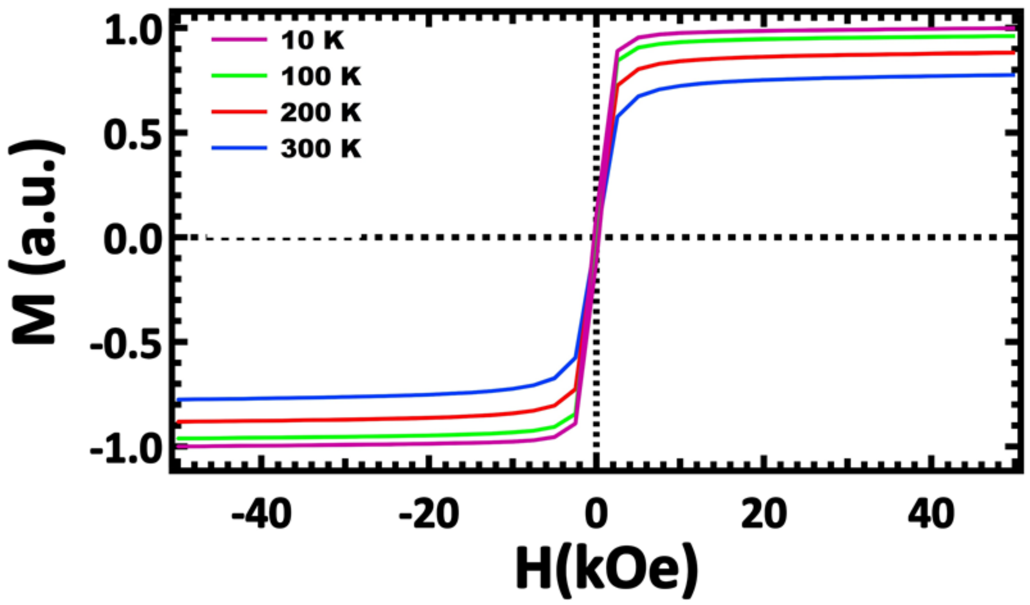

Figure 2. Sample magnetization vs. applied magnetic field as a function of temperature.

2.3. Inter-particle Magnetic Correlation Length

Spin-flip (↑↓ + ↓↑) SANS scattering at 0.005 T and 200 K yields the magnetic-only scattering [6, 7, 8] of the nanoparticles at near remanence, Fig. 3. The lack of a (111) FCC reflection, as observed for the structural scattering in Fig. 1, indicate that the nanoparticles are not magnetically correlated over long length scales at remanence. However, the data cannot be fit with a simple core or core|shell model which assumes that the nanoparticles are magnetically uncorrelated (black dashed curve). Rather the data require a model that includes magnetic correlations between the nanoparticles (solid blue curve). Here we use a fractal model consisting of correlated spheres [12] to evaluate this correlation length, ϵ. The fractal dimension, d, of spherical nanoparticles is 2 [13]. The nanoparticle diameter is fit at (7.31 ± 0.04) nm, which agrees with the transmission electron microscopy average of 7.4 nm [5], and $\epsilon = (4.27 \pm 0.06)$ nm. The Guinier radius of a cluster, R_G, is given by [12],

$$R_G^2 = d(d+1)\epsilon^2/2. \quad (2)$$

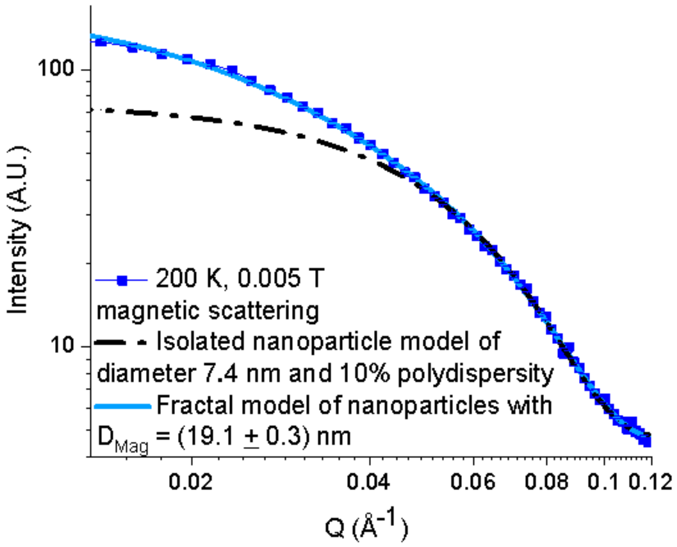

Figure 3. Spin-flip small-angle neutron scattering arises from the scattering of magnetic moments, but not from structural scattering, within the scattering sample. Such scattering, thus, measures the magnetic scattering from individual nanoparticles plus any magnetic correlations that form between the nanoparticles. A small magnetic guide filed of 0.005 T was used to retain the neutron spin polarization.

Assuming the magnetic correlation is spherical, then its radius, R_{Mag}, is related to R_G as [13]

$$R_{Mag}^2 = \frac{5}{3} R_G^2. \tag{3}$$

This sets R_{Mag} at (9.55 ± 0.14) nm, or $D_{Mag} = (19.1 \pm 0.3)$ nm.

It is worthwhile to note that the D_{Mag} at remanence is roughly one-half D_{Struc} for this sample. Thus, it is likely for our primary sample that the magnetic correlation is similarly reduced from the structural coherence of $\sqrt(3)L$ or n.n.n.n, Table I. of the main text. Therefore, we leave the determination of how many of the n.n.n.n. to include in the calculation of J as the only fitting parameter.

3. Statistical Significance of Observed Magnon Peaks

For weak magnon signals coincident with large background scattering, the most reliable method of extracting the magnons is through subtraction of one data set from another, varied in either temperature or magnetic field. The uncertainty (σ = one sigma) from the unsubtracted data sets is calculated as the square root of the total counts measured, and the uncertainty of the subtraction from data sets 1 and 2 is determined in the usual manner as $\sigma_{subtraction} = \sqrt{\sigma_1^2 + \sigma_2^2}$. With this treatment it becomes apparent that the signal observed is not significantly larger than the statistical fluctuations. Thus, we shall apply Akaike Information Criteria (AIC) [14] and Bayesian Information Criteria

(BIC) [15] tests to determine whether the data is statistically robust and most likely to be correctly associated with a Gaussian-like peak or if the data could be equally well represented by a sloping background (linear or exponential), which would be a null result. Residual sum of squares (RSS) minimization of model fits and the AIC, BIC statistical comparison between the model fits were performed using OriginPro 2017 [2].

Although neither the AIC nor the BIC approaches can be used to determine if any given model correctly represents the data, they can be used effectively to compare models on the same data set; comparatively lower AIC and BIC values correspond to models that are more likely to accurately represent the data. Specifically, the AIC and BIC values are determined as,

$$AIC = 2k + n \cdot ln(RSS/n)C \tag{4}$$

$$BIC = n \cdot ln(RSS/n) + k \cdot ln(n), \tag{5}$$

where k is the number of fitted parameters, n is the number of data points, and C is a data-set specific constant that drops out in the difference of AIC values. If the number of fitted parameters between models is the same, then the lowest AIC simply selects for the model with the lowest RSS. The relative likelihood of model 1 compared to model 2 in the AIC test is determined from $e^{(AIC_2 - AIC_1)/2}$. For the BIC test, absolute BIC differences of less than 2 are considered inconclusive, while those of 2 or more are considered to positively select one model over the other. Both AIC and BIC analysis select for a reduction of fitting parameters along with a reduction in RSS, with the penalty of extra parameters typically being larger in the BIC test.

3.1. ***Q=0.16*** $\mathring{A}^{-1}$ ***Data***

A shift of extra intensity along the energy axis is observed between 0 T, 4 T, and 8 T (Fig. 3a of the main text). To extract a magnon peak the 0 T data is subtracted from the 4 T data, resulting in Fig. 4 (comparable to Fig. 3b of the main text, but without any shift in energy to minimize data processing). We note we cannot extend the fit beyond -0.4 meV since the 0 T peak has weight here and causes the difference to become negative. Models of a Gaussian peak versus linear background (null result) are compared. Both the AIC and the BIC statistical analysis select the Gaussian peak as the preferred model, with the AIC approach indicating a Gaussian peak is more statistically likely than a linear fit by a factor of 1.35. The exponential model does not fit the data well and is excluded from further consideration.

This Gaussian-like peak becomes more apparent by eye (even though the information content is not increased) if the data are rebinned every two points, where A and B rebins represent each of the two possible ways the data could be combined, Fig. 5.

The intensity difference from the 8 T 0 T data sets is more spread out in energy and is not statistically significant as a peak by itself, Fig. 6. AIC test selects the lower parameter linear fit, while the BIC test is inconclusive. However, knowing that the 4 T

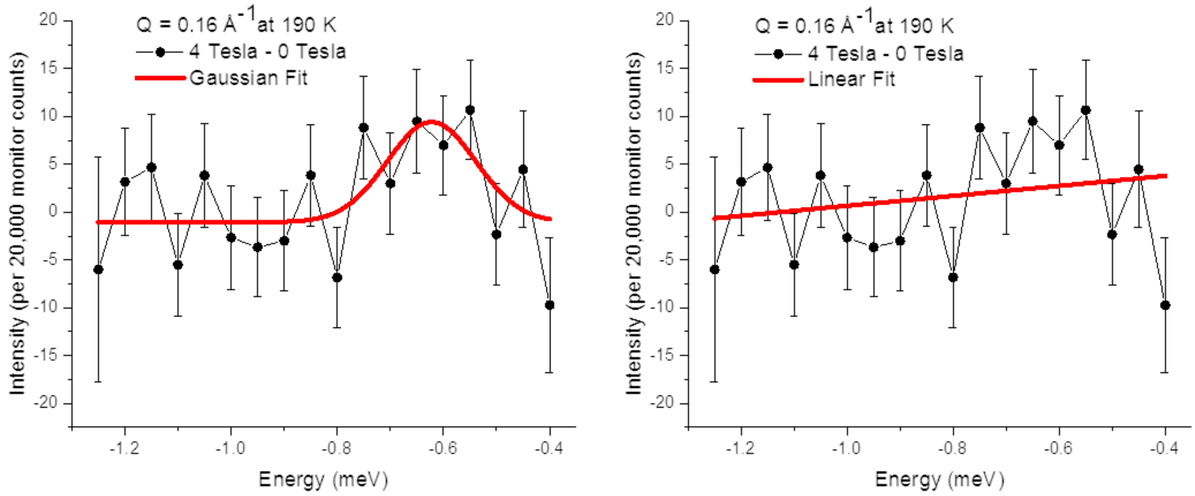

Figure 4. Gaussian and linear models fits at Q = 0.16 $\AA^{-1}$. Both AIC and BIC tests select for the Gaussian peak.

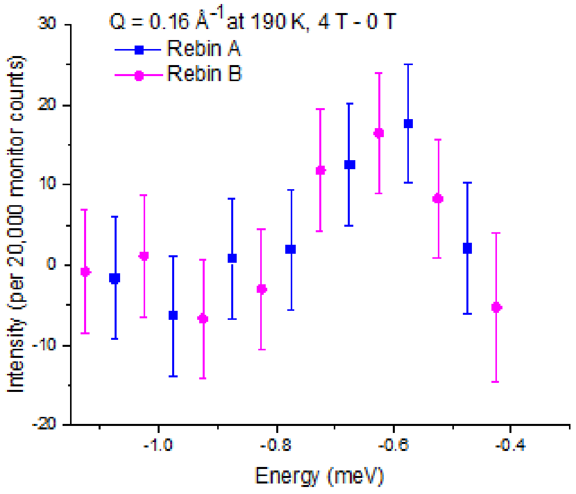

Figure 5. Rebinning the data every two points at Q = 0.16 $\AA^{-1}$, where Rebin A and B indicate the two possible ways to do this, yields a prominent peak.

data contains a Gaussian-like magnon, we can still use the 8 T 0 T data set to gauge the amount the peak shifts in energy with applied magnetic field.

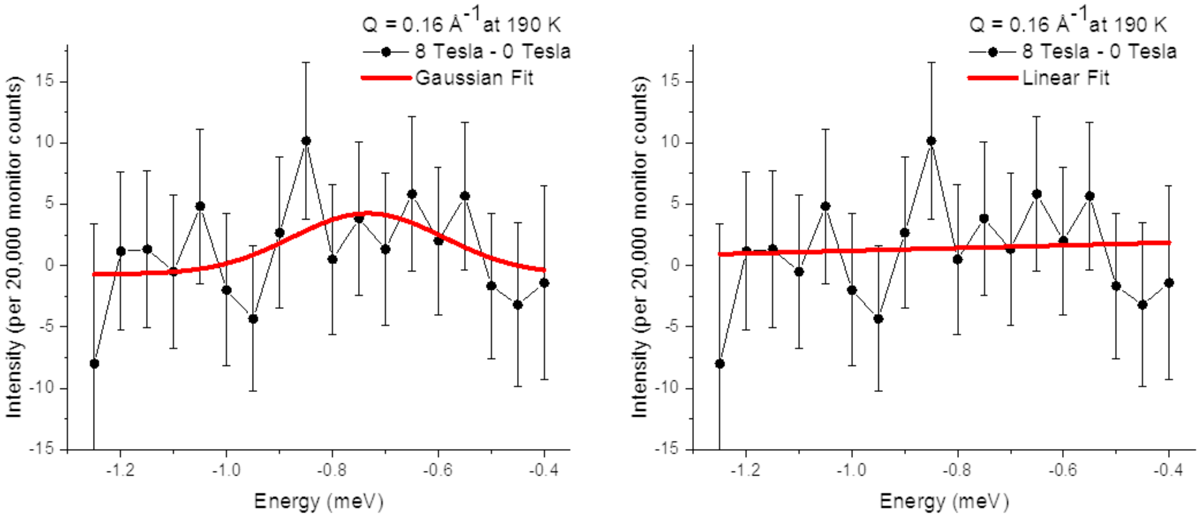

Figure 6. Gaussian and linear models fits at Q = 0.16 $\AA^{-1}$ for the 8 T - 0 T data. AIC test selects for the lower-parameter linear fit, while the BIC test is inconclusive.

3.2. ***Q=0.20*** $\AA^{-1}$ ***Data***

The Q = 0.20 $\AA^{-1}$ data set is challenging to model since the peak of interest is situated on the edge of the dominant, quasi-elastic Bragg peak. Note that the quasi-elastic peak is larger at 21 K than at 300 K (Fig. 2a of the main text) such that all the subtracted data points can be utilized up to the point at which a large, negative low-Q difference persists (as done here, Fig. 7). The AIC test selects the Gaussian peak model over the linear fit by a factor of 2.0 and over the exponential fit by a factor of 7.3. The BIC tests are inconclusive. Similar results are obtained even if the one or two points closest to the quasi-elastic peak are removed.

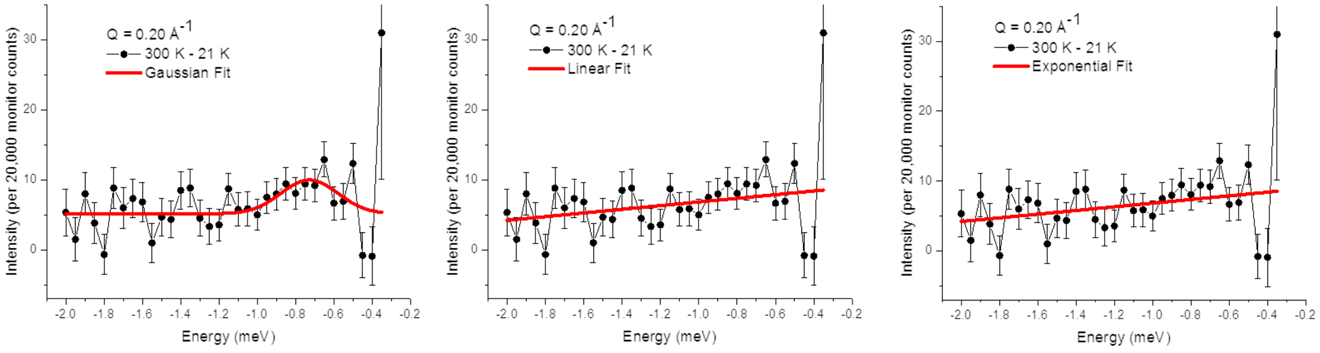

Figure 7. Gaussian, linear, and exponential models fits at Q = 0.20 $\AA^{-1}$. The AIC test selects for the Gaussian model, while the BIC test is inconclusive in the comparison between the Gaussian and linear fits (exponential fit is not preferred).

3.3. ***Q=0.25*** $\AA^{-1}$ ***Data***

Although our Inter-particle Magnon Model suggests that two magnon peaks could be present in the data, only the highest Q peak is distinctive enough to warrant a statistical evaluation due to the prominence of the sloping background resulting from the difference between 300 K and 21 K. Thus, we fit the data with a Gaussian peak plus linear slope, a linear slope, and an exponential slope, Fig. 8. (Note we do not further discuss the Gaussian peak plus exponential slope model since this returns a linear-like background slope that has the same fit quality as the Gaussian plus linear model, but with additional fit parameters.) Although the BIC test is inconclusive, the AIC test selects the Gaussian plus linear model over the linear or exponential fits by factors of 1.4 and 1.9, respectively.

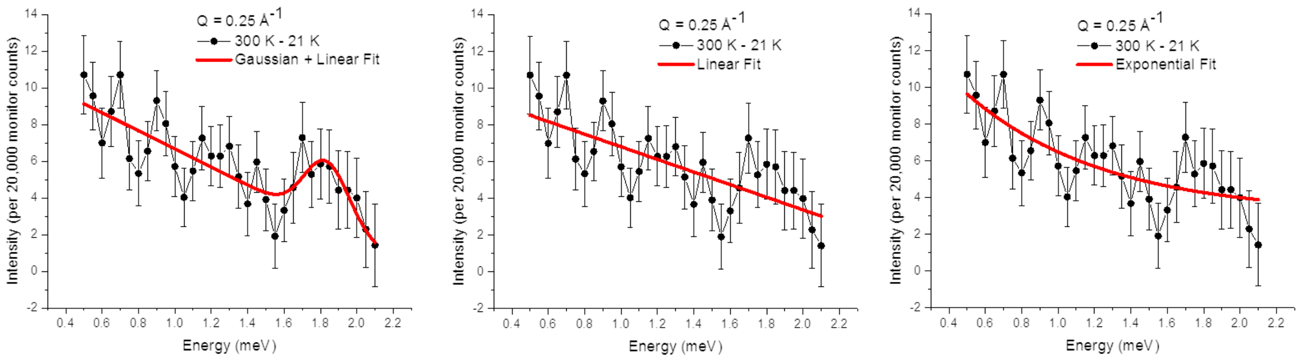

Figure 8. Gaussian + linear, linear, and exponential models fits at Q = 0.25 $\AA^{-1}$. The AIC test selects for the Gaussian + linear model, while the BIC test is inconclusive.

Alternatively, it is common practice to fit the background from one range, and then model the peak after subtraction of this measured slope in another range. Here we fit the data from 0.5 meV to 1.0 meV with a linear slope, and subtract this from the entire data set, Fig. 9. In this case both AIC and BIC tests select the Gaussian peak over a linear slope, the AIC test by a factor of 27.9.

3.4. ***Summary of Statistical Significance***

Statistical analysis indicates that the statistically significant Gaussian-like peaks have been found with the AIC test at Q = 0.16 $\AA^{-1}$ (4 T 0 T), Q = 0.20 $\AA^{-1}$ (300 K 21 K), and Q = 0.25 $\AA^{-1}$ (300 K 21 K, highest Q peak) and with the BIC test at Q = 0.16 $\AA^{-1}$ (4 T 0 T) and Q = 0.25 $\AA^{-1}$ (300 K 21 K, manual background removal, highest Q peak). These are summarized in Table S1. We note that the lower-Q, secondary peak at = 0.25 $\AA^{-1}$ is questionable and can neither be statistically confirmed nor excluded based on the data. Although individual magnon peaks are weak and suffer from significant statistical noise, in aggregate they are statistically relevant. Taken together, they are well described by the Inter-particle Magnon Model presented in the main text.

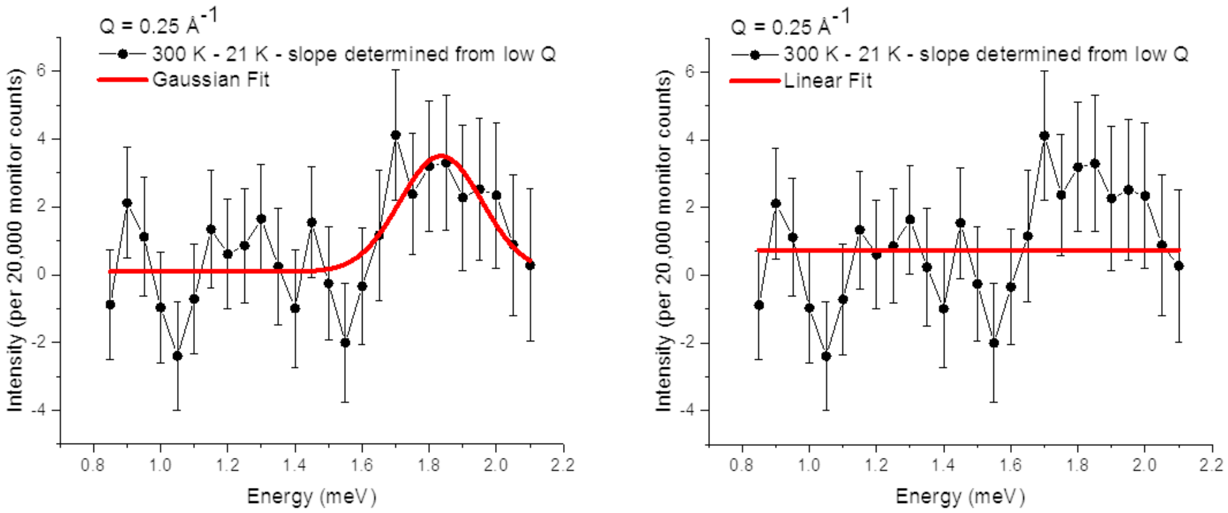

Figure 9. Gaussian and linear models fits at Q = 0.25 $\AA^{-1}$ after manual background removal has been performed from fitting the 0.5 meV to 1.0 meV region with a linear slope. Both AIC and BIC tests select for the Gaussian model.

Table 1. Comparative fit results *Indicates that the model is preferred by a statistically significant amount. k is the number of fit parameters.

Models for a given Q	AIC Value	BIC Value
Q = 0.16 $\AA^{-1}$ (4 T - 0 T):		
Gaussian (peak, k = 4)	*7.63349	*7.08535
Linear (null, k = 2)	8.22756	9.18439
Q = 0.20 $\AA^{-1}$ (300 K - 21 K):		
Gaussian (peak, k = 4)	*12.65742	18.14637
Linear (null, k = 2)	14.05163	17.83072
Exponential (null, k = 3)	16.63095	21.35708
Q = 0.25 $\AA^{-1}$ (300 K - 21 K):		
Gaussian + Linear (peak, k = 5)	*-4.19698	1.55129
Linear (null, k = 2)	-3.46573	0.19621
Exponential (null, k = 3)	-2.88251	1.67495
Q = 0.25 $\AA^{-1}$ (300 K - 21 K, manual background removal):		
Gaussian (peak, k = 4)	*-6.95694	*-3.66645
Linear (null, k = 2)	-0.30018	1.69428

4. Zeeman Excitation Shift at Q = 0.16 $\AA^{-1}$

At Q = 0.16 $\AA^{-1}$ the magnon increases linearly in energy with applied field between 0 T and 8 T due to the Zeeman energy contribution. In order to get a mid-point fit for the peaks at 4 T and 8 T, Fig. 3 of the main text, the background slope (shown) was subtracted from each data set, Fig. 10. The resulting 4 T peak spans -0.75 meV to

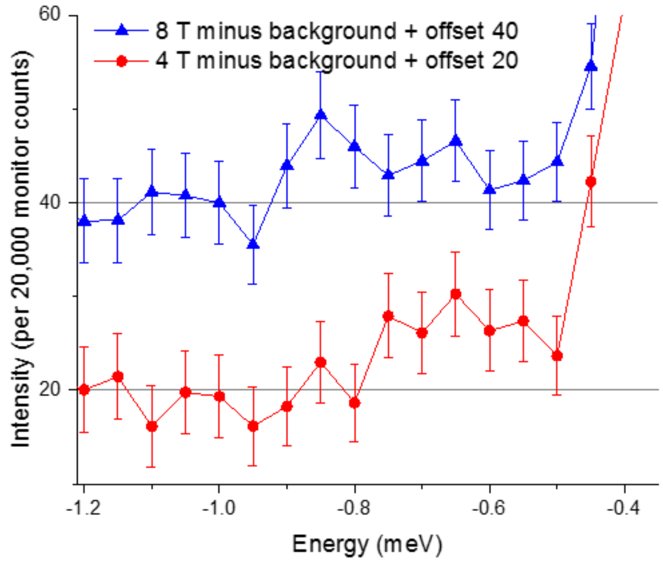

[h]

Figure 10. The 8 T and 4 T raw data minus the plotted background slope from Fig. 3 of the main text are plotted here. Offsets of 40 and 20, respectively, are retained.

-0.50 meV, with an intensity-weighted average of -0.638 meV. The resulting 8 T peak spans -0.90 meV to -0.65 meV, with an intensity-weighted average of -0.778 meV. Thus, the shift between 4 T and 8 T is -0.14 meV with an uncertainty of about ± 0.02 meV.

5. Temperature Conversion

The Q = 0.16 $\AA^{-1}$ data was measured at 190 K, while the Q = 0.20 $\AA^{-1}$ and 0.25 $\AA^{-1}$ data sets were measured at 300 K. The excitation energy is $\propto$ the sample magnetization squared, M^2. As discussed iabove, $\left(\frac{M_{190\ K}}{M_{300\ K}} = 1.17\right)^2 = 1.37$. This is the formula used to determine excitation values at 190 K relative to those calculated at 300 K.

6. Dzyaloshinskii-Moriya Interaction

Since a non-zero Q_0 shift, Eqn. 1 of the main text, is required in order to avoid a non-physical negative energy gap, one must consider the possibility that a symmetry-breaking interaction such as the Dzyaloshinskii-Moriya (DM) interaction [16] is involved. Additionally, it has been shown that the DM interaction could be responsible for canted shell structures within magnetite core|manganese ferrite shell nanoparticles [5] and Fe_3O_4 nanoparticles [17]. If the DM interaction was responsible for $Q_0 \neq 0$, then the measured spin stiffness of 51 meV $\AA^2$ ± 12 meV $\AA^2$ would be intra-particle and reflect the atomic exchange of the nanoparticle, which is considerably lower than either the spin stiffness of bulk $MnFe_2O_4$ at 173 meV $\AA^2$ [18] or bulk Fe_3O_4 at 233 meV $\AA^2$ [19]. In addition, the value of Q_0 is set by $-DM/a\chi$ [20], where a is the lattice constant of 8.396 $\AA$ [5], DM is the interatomic Dzyaloshinskii-Moriya interaction strength, and χ is

the interatomic exchange strength. Using the atomistic simulation, VAMPIRE, it was determined that with χ set equal to the average interatomic exchange between Fe-Fe and Mn-Fe in bulk $MnFe_2O_4$, the minimum ratio of DM to χ which produced shell canting within magnetite core|manganese ferrite shell nanoparticles would be 1.8 [5]. Similarly, high resolution electron loss spectrocopy on Fe_3O_4 nanoparticles combined with the density functional theory program, WIEN NCN, determined that although χ was reduced in nanoparticle form, the DM to χ ratio required to reproduce the observed canted shell formation remains at 1.8 [17]. Both approaches, spanning the range from bulk-like to suppressed χ values, produce similar DM to χ ratios required for shell canting and result in a Dzyaloshinskii-Moriya interaction Q_0 shift of ≈ 0.21 $\AA^{-1}$ [20]. Thus, the experimentally observed Q_0 shift of 0.0714 $\AA^{-1}$ can be far better explained as renormalization of q by nearest reciprocal-lattice vector arising from nanoparticle-to-nanoparticle spacing within their ordered lattice than by the DM interaction.

7. Lattice Truncation

As discussed in the main text, only ferromagnetic (FM) coupling between the central nanoparticle and neighboring nanoparticles is considered. For $M \perp$ plane calculations this involves coupling between the central nanoparticle and the 2nd and 3rd layers (2 n.n., 2 n.n.n., and 3 n.n.n.n.), while for $M \parallel$ plane calculations this involves coupling between the central nanoparticle and the 1st and 2nd layers (1 n.n., 2 n.n.n., 1 n.n.n.n., and 2 n.n.n.n.). However, since the magnetic domains are likely reduced from the structural crystallites with coherence up to n.n.n.n. (see above), the number of n.n.n.n. to include in the magnetic calculations is a fitting parameter. Table II. of the main text shows that the 3 n.n.n.n. have strong magnetic coupling to the central nanoparticle (especially for $M \perp$ plane), while 1 n.n.n.n. and 2 n.n.n.n. have significantly weaker magnetic coupling to the central nanoparticle. Thus, the calculations of J_NS that follow will include all possible n.n. and n.n.n. plus the 3 n.n.n.n., but none of the 1 n.n.n.n. or 2 n.n.n.n., unless otherwise stated. Neighbor nanoparticle positions are listed in Table I. of the main text.

8. Magnon Calculation for $\hat{Q}$ along [1,1,0] and Equivalent Directions

For (110) and equivalent reflections, $Q_0 = 0.124$ $\AA^{-1}$. The excitations resulting from the combination of Eqn. 2 and Table I. of the main text are:

$$\hbar\omega(1\ n.n.)A_1 = J_N S\left[6A_1 - 4A_2 cos(Lq/2) - 2A_2 cos(Lq)\right]$$
$$\hbar\omega(2\ n.n.)A_1 = J_N S\left[6A_1 - 2A_2 - 4A_2 cos(Lq/2)\right]$$
$$\hbar\omega(2\ n.n.n.)A_1 = J_N S\left[6A_1 - 2A_2 - 4A_2 cos(Lq)\right]$$
$$\hbar\omega(1\ n.n.n.n.)A_1 = J_N S\left[6A_1 - 2A_2 - A_2 4cos(3Lq/2)\right]$$
$$\hbar\omega(2\ n.n.n.n.)A_1 = J_N S\left[12A_1 - A_2 4cos(Lq/2) - A_2 4cos(3Lq/2) - A_2 4cos(Lq)\right]$$
$$\hbar\omega(3\ n.n.n.n.)A_1 = J_N S\left[6A_1 - 2A_2 - 4A_2 cos(Lq/2)\right]$$

Table 2. Ferromagnetic acoustic excitations for $\hat{Q} = [1, 1, 0]$

Q ($\AA^{-1}$)	q ($\AA^{-1}$)	Temperature (K)	M $\perp$ plane (meV)	M $\parallel$ plane (meV)
0.16	0.036	300 $\rightarrow$ 190	0.051 $\rightarrow$ 0.070	0.072 $\rightarrow$ 0.099
0.20	0.076	300	0.23	0.32
0.25	0.126	300	0.62	0.88

Table 3. Ferromagnetic acoustic excitations for $\hat{Q} = [1, 0, 0]$

Q ($\AA^{-1}$)	q ($\AA^{-1}$)	Temperature (K)	M $\perp$ plane (meV)	M $\parallel$ plane (meV)
0.16	0.089	300 $\rightarrow$ 190	0.31 $\rightarrow$ 0.42	0.43 $\rightarrow$ 0.59
0.20	0.129	300	0.65	0.91
0.25	0.179	300	1.25	1.76

Acoustic ($A_i = A_j$) excitation values are calculated by inserting the dipolar coupling constants (J_NS) from Table II of the main text into the equations above: These excitations are smaller than the experimentally observed energies, Fig. 4b of the main text. Even if all of the possible n.n.n.n. were included (*i.e.* adding 1 n.n.n.n. and 2 n.n.n.n.), $M \parallel$ plane excitation energies would only increase to (0.15, 0.50, and 1.36) meV at Q = (0.16, 0.20, and 0.25) $\AA^{-1}$ at 300 K, which is still too small account for the experimental results. Changing the number of included n.n.n.n. has no effect on $M \perp$ plane calculations.

9. Magnon Calculation for $\hat{Q}$ along [1,0,0] and Equivalent Directions

For (100) and equivalent reflections, $Q_0 = 0.0714\ \AA^{-1}$. The excitations resulting from the combination of Eqn. 2 and Table I. of the main text are:

$$\hbar\omega(1\ n.n.)A_1 = J_N S\left[6A_1 - 2A_2 - 4A_2 cos(\sqrt{3}Lq/2)\right]$$

$$\hbar\omega(2\ n.n.)A_1 = J_N S\left[6A_1 - 2A_2 cos(Lq/\sqrt{(3)}) - 4A_2 cos(Lq/\sqrt{(12)})\right]$$

$$\hbar\omega(2\ n.n.n.)A_1 = J_N S\left[6A_1 - 2A_2 cos(2Lq/\sqrt{(3)}) - 4A_2 cos(Lq/\sqrt{3})\right]$$

$$\hbar\omega(1\ n.n.n.n.)A_1 = J_N S\left[6A_1 - 2A_2 cos(\sqrt{(3)}Lq) - A_2 4cos(3Lq/2)\right]$$

$$\hbar\omega(2\ n.n.n.n.)A_1 = J_N S\left[12A_1 - 4A_2 cos(Lq/\sqrt{12}) - 4A_2 cos(5Lq/\sqrt{12}) - 4A_2 cos(2Lq/\sqrt{3})\right]$$

$$\hbar\omega(3\ n.n.n.n.)A_1 = J_N S\left[6A_1 - 2A_2 cos(Lq/\sqrt{(3)}) - 4A_2 cos(Lq/\sqrt{(12)})\right]$$

Acoustic ($A_i = A_j$) excitation values are calculated by inserting the dipolar coupling constants (J_NS) from Table II of the main text into the equations above:

The FM excitations are close to the experimentally observed energies, Fig. 4b of the main text. If we drop the 3 n.n.n.n., the $M \perp$ plane excitation energies decrease

Table 4. Ferromagnetic acoustic excitations for $\hat{Q} = [0, 0, 1]$

Q ($\AA^{-1}$)	q ($\AA^{-1}$)	Temperature (K)	M $\perp$ plane (meV)	M $\parallel$ plane (meV)
0.16	0.084	300 $\rightarrow$ 190	1.58 $\rightarrow$ 2.16	0.023 $\rightarrow$ 0.032
0.20	0.124	300	3.42	0.050
0.25	0.174	300	6.69	0.097

slightly to (0.35, 0.54, and 1.04) meV at Q = (0.16, 0.20, and 0.25) $\AA^{-1}$ at 300 K, which doesn't fit the experimentally observed energies quite as well. Conversely, including all possible n.n.n.n. increases $M \parallel$ plane excitation energies to (1.06, 1.62, and 3.11) meV at Q = (0.16, 0.20, and 0.25) $\AA^{-1}$ at 300 K, which no longer fits the data. Thus, including the strongest 3 n.n.n.n. interactions, but not the weaker 1 n.n.n.n. and 2 n.n.n.n. interactions, best describes the experimental data, Fig. 4b of the main text.

10. Magnon Calculation for $\hat{Q}$ along [0,0,1] and Equivalent Directions

For (002) and equivalent reflections, $Q_0 = 0.0757\ \AA^{-1}$. The excitations resulting from the combination of Eqn. 2 and Table I. of the main text are:

$$\hbar\omega(1\ n.n.)A_1 = J_N S\left[6A_1 - 6A_2\right]$$
$$\hbar\omega(2\ n.n.)A_1 = J_N S\left[6A_1 - 6A_2 cos(Lq\sqrt{(\frac{2}{3})})\right]$$
$$\hbar\omega(2\ n.n.n.)A_1 = J_N S\left[6A_1 - 6A_2 cos(Lq\sqrt{(\frac{2}{3})})\right]$$
$$\hbar\omega(1\ n.n.n.n.)A_1 = J_N S\left[6A_1 - 6A_2\right]$$
$$\hbar\omega(2\ n.n.n.n.)A_1 = J_N S\left[12A_1 - 12A_2 cos(Lq\sqrt{(\frac{2}{3})})\right]$$
$$\hbar\omega(3\ n.n.n.n.)A_1 = J_N S\left[6A_1 - 6A_2 cos(Lq\sqrt{(\frac{8}{3})})\right]$$

Acoustic ($A_i = A_j$) excitation values are calculated by inserting the dipolar coupling constants ($J_N S$) from Table II of the main text into the equations above:

The M $\perp$ plane excitations are significantly larger than the experimentally observed energies, Fig. 4b of the main text. If none of the n.n.n.n. were included, the $M \perp$ plane excitation energies would decrease to (1.18, 1.86, and 3.68) meV at Q = (0.16, 0.20, and 0.25) $\AA^{-1}$ at 300 K, which is still too large to describe the experimental data.

11. Optical Excitations

Optical excitations, $A_i = -A_j$, can be ruled out from explaining the data presented since they form nearly Q-independent excitations within the Q-range of interest with large gaps on the order to 10's of meV to 100's of meV. As an example, the FM 1 n.n. with $M \parallel$ plane and $\hat{Q}$ = [1,0,0] would produce excitations of (132.47, 131.65, and 131.22) meV at Q's of (0.16, 0.20, 0.25) $\AA^{-1}$ at 300 K.

References

[1] http://www.sasview.org/

[2] The identification of any commercial product or trade name does not imply endorsement or recommendation by the National Institute of Standards and Technology.

[3] S. R. Kline, J. Appl. Cryst. 39, 895 (2006).

[4] B. E. Warren, X-Ray Diffraction, Dover Publications, INC., 251-254 (1969).

[5] Samuel D. Oberdick, Ahmed Abdelgawad, Carlos Moya, Samaneh Mesbahi-Vasey, Demie Kepaptsoglou, Vlado K. Lazarov, Richard F. L. Evans, Daniel Meilak, Elizabeth Skoropata, Johan van Lierop, Ian Hunt-Isaak, Hillary Pan, Yumi Ijiri, Kathryn L. Krycka, Julie A. Borchers, Sara A. Majetich, Scientific Reports, 8, 3425 (2018).

[6] A. Michels and J.Weissmuller, Rep. Prog. Phys. 71, 066501 (2008).

[7] K. L. Krycka, R. A. Booth, C. R. Hogg, Y. Ijiri, J. A. Borchers, W. C. Chen, S. M.Watson, M. Laver, T. R. Gentile, L. R. Dedon, S. Harris, J. J. Rhyne, and S. A. Majetich, Phys. Rev. Lett. 104, 207203 (2010).

[8] W. Schweika, J. Phys. Conf. Ser. 211, 012026 (2010).

[9] K. Krycka, W. Chen. J. Borchers, and S. Watson, J. Appl. Cryst. 45, 546-553 (2012).

[10] H. Matsuoka, H. Tanaka, T. Hashimoto and N. Ise, Phys. Rev. B, 36, 1754-1765 (1987)

[11] H. Matsuoka, H. Tanaka, N. Iizuka, T. Hashimoto and N. Ise, Phys. Rev. B, 41, 3854-3856 (1990)

[12] J. Teixeira, J. Appl. Cryst., 21, 781-785 (1988).

[13] The SANS Toolbox: https://www.ncnr.nist.gov/staff/hammouda/the_sans_toolbox.pdf.

[14] H. Akaike, IEEE Transactions on Automatic Control, 19 (6): 716723 (1974)

[15] G. E. Schwarz, Annals of Statistics, 6 (2): 461464 (1978)

[16] M. Garst, J. Waizner, and D. Grundler, Journal of Physics D: Applied Physics, 50(29), 293002 (2017).

[17] D. S. Negi, H. Sharona, U. Bhat, S. Palchoudhury, A. Gupta,and R. Datta, Phys. Rev. B 95, 174444 (2017).

[18] V. C. Rakhecha, L. Madhav Rao, N. S. Satya Murthy, and B. S. Srinivasan, Phys. Lett. 40A, 101 (1972).

[19] H. A. Alperin, O. Steinsvoll, R. Nathans, and G. Shirane, Phys. Rev. 154, 508 (1967).

[20] T. J. Sato, D. Okuyama, T. Hong, A. Kikkawa, Y. Taguchi, T. H. Arima, and Y. Tokura, Phys. Rev. B, 94(14), 144420 (2016).
